# Deciphering Network Crosstalk: The Current Status and Potential of miRNA Regulatory Networks on the HSP40 Molecular Chaperone Network

**DOI:** 10.3389/fgene.2021.689922

**Published:** 2021-06-21

**Authors:** Lion Budrass, Richard P. Fahlman, Sue-Ann Mok

**Affiliations:** ^1^Neuroscience and Mental Health Institute, University of Alberta, Edmonton, AB, Canada; ^2^Department of Biochemistry, University of Alberta, Edmonton, AB, Canada; ^3^Department of Oncology, University of Alberta, Edmonton, AB, Canada

**Keywords:** J-proteins, Hsp40, microRNAs, chaperone, target prediction

## Abstract

Molecular chaperone networks fulfill complex roles in protein homeostasis and are essential for maintaining cell health. Hsp40s (commonly referred to as J-proteins) have critical roles in development and are associated with a variety of human diseases, yet little is known regarding the J-proteins with respect to the post-transcriptional mechanisms that regulate their expression. With relatively small alterations in their abundance and stoichiometry altering their activity, post-transcriptional regulation potentially has significant impact on the functions of J-proteins. MicroRNAs (miRNAs) are a large group of non-coding RNAs that form a complex regulatory network impacting gene expression. Here we review and investigate the current knowledge and potential intersection of miRNA regulatory networks with the J-Protein chaperone network. Analysis of datasets from the current version of TargetScan revealed a great number of predicted microRNAs targeting J-proteins compared to the limited reports of interactions to date. There are likely unstudied regulatory interactions that influence chaperone biology contained within our analysis. We go on to present some criteria for prioritizing candidate interactions including potential cooperative targeting of J-Proteins by multiple miRNAs. In summary, we offer a view on the scope of regulation of J-Proteins through miRNAs with the aim of guiding future investigations by identifying key regulatory nodes within these two complex cellular networks.

## Introduction

MicroRNA (miRNA) networks of gene regulation and molecular chaperone networks both consist of complex webs of interactions with broad implications in shaping the proteome. In both networks, the activity of individual molecules impacts many target or client molecules, resulting in broad regulation of protein homeostasis (proteostasis) (Hipp et al., 2014). Here, we discuss the intersection of these two major cellular networks. Specifically, we review the current reported miRNA regulatory interactions on the HSP40 family of chaperones, commonly referred to as J-proteins. Furthermore, we will consider the potential expanded network of interactions predicted by bioinformatic analysis. For this study, we will focus on J-proteins which represent the largest and most diverse group of molecular chaperones (Kampinga and Craig, 2010).

## Molecular Chaperones

The cellular proteostasis network coordinates protein synthesis, degradation, and stress responses to ensure the correct folding, concentration, and localization of proteins to effectively carry out their cellular functions (Hipp et al., 2014). Molecular chaperones are an integral component of all proteostasis processes. They are a large group of ~300 proteins (Brehme et al., 2014) that operate to recognize and deal with protein misfolding issues arising throughout the proteome. A multitude of functions is attributed to individual chaperone members with respect to facilitating protein folding, protein disaggregation, sequestration of aggregates, and directing misfolded proteins toward cellular degradation pathways (Kim et al., 2013; Kaushik and Cuervo, 2018; Nillegoda et al., 2018). Individual chaperones can often be grouped into distinct protein families e.g., J-proteins, Hsp60, Hsp70, Hsp90, sHsp. The most well-studied mechanistic aspect of chaperone activities is their ability to reversibly bind, and release unfolded and misfolded substrates (often termed clients) to promote their proper folding and prevent aggregation. Members of the major Heat shock protein 70 (Hsp70) and Heat shock protein 90 (Hsp90) families interact with hundreds of client proteins (Kerner et al., 2005; Taipale et al., 2012). In contrast, some chaperones members of the J-protein family show evidence of more discrete client binding profiles (Fotin et al., 2004; Gong et al., 2009; Kakkar et al., 2016a,b; Craig and Marszalek, 2017). However, chaperone interactions are not limited to client binding. Chaperones are known to work cooperatively with other chaperones and components of the proteostasis network, functioning as larger protein complexes (Taipale et al., 2014; Rizzolo et al., 2017; Freilich et al., 2018; Karunanayake and Page, 2021). For example, HSP70s have ATPase activities that allow for cycling between client binding and release—J-protein and Bag chaperone families strongly stimulate this cycling by promoting ATP hydrolysis and release, respectively (Freilich et al., 2018). Another factor contributing to the complex nature of chaperone activities is the different subcellular localization and expression levels of individual chaperones that directly influence client binding and stoichiometry of formed chaperone complexes (Craig and Marszalek, 2017).

### The J-Protein Family of Chaperones. Potential for Regulation to Shape Cellular Proteostasis

The modular nature of chaperone complexes is proposed to contribute to the fine-tuning of chaperone recruitment and processing of specific clients in the cell. One large family of chaperones that facilitate modularity is the J-protein family. There are over 40 identified J-protein family members in humans (Kampinga and Craig, 2010), which are listed in Table 1. All members share a characteristic J-domain that facilitates modes of Hsp70 binding and ATP hydrolysis (Karzai and McMacken, 1996; Jiang et al., 2007; Kityk et al., 2018; Faust et al., 2020) and thus, are commonly referred to as J-proteins. However, beyond this shared domain, there is incredible diversity between J-protein members with respect to their structural and functional domains: individual J-proteins possess unique combinations of different client binding domains, localization signals, and enzymatic activities (Kampinga and Craig, 2010). J-proteins are roughly classified into three groups by structure. The A-class J-proteins share an overall domain structure similar to the *E.coli* J-proteins whereas the B-class J-proteins have only partially retained these domains. The remaining J-proteins are simply categorized as C-class (Kampinga and Craig, 2010).

**Table 1:** J-proteins and their validated miRNA targeting.

**Hsp40**	**Targeting miRNA**	**References**	**Hsp40**	**Targeting miRNA**	**References**
DNAJA1	-	-	DNAJC6	-	-
DNAJA2	-	-	DNAJC7	-	-
DNAJA3	-	-	DNAJC8	-	-
DNAJA4	-	-	DNAJC9	-	-
DNAJB1	miR-370, miR-543	Evert et al., 2018	DNAJC10	-	-
DNAJB2a	-	-	DNAJC11	-	-
DNAJB2b	-	-	DNAJC12	-	-
DNAJB4	-	-	DNAJC13	-	-
DNAJB5	-	-	DNAJC14	-	-
DNAJB6a	-	-	DNAJC15	-	-
DNAJB6b	-	-	DNAJC16	-	-
DNAJB7	-	-	DNAJC17	-	-
DNAJB8	-	-	DNAJC18	-	-
DNAJB9	miR-25-32-92-363-367 family	Wang et al., 2020	DNAJC19	-	-
DNAJB11	miR-29b	Beitzinger et al., 2007	DNAJC20	-	-
DNAJB12	miR-148-152 family	Ma et al., 2020	DNAJC21	-	-
DNAJB13	-	-	DNAJC22	-	-
DNAJB14	-	-	DNAJC23	-	-
DNAJC1	-	-	DNAJC24	-	-
DNAJC2	-	-	DNAJC25	-	-
DNAJC3	miR-200 family	Belgardt et al., 2015	DNAJC26	-	-
DNAJC4	-	-	DNAJC27	-	-
DNAJC5	-	-	DNAJC28	-	-
DNAJC5B	-	-	DNAJC29	-	-
DNAJC5G	-	-	DNAJC30	-	-

Multiple J-proteins can compete for interactions with the same Hsp70. Therefore, varying the expression levels of individual J-proteins could, in turn, fine-tune the proteostasis of specific client subsets in a cell. Furthermore, there is evidence that changes in the balance of chaperone concentrations can have significant effects on chaperone complex formation and function (Kanelakis et al., 1999; Kundrat and Regan, 2010; Cabrera et al., 2019). For example, increasing the cellular concentrations of the mitochondrial J-protein, DNAJA3, interferes with the ability of the mitochondrial Hsp70 to bind substrates resulting in protein aggregation and mitochondria fragmentation (Lee B. et al., 2015). More broadly, J-proteins may be used to modulate chaperone network function to deal with the specific proteomes of different tissues (Uhlén et al., 2015). Indeed, individual J-proteins do show variations in tissue-specific expression (Hageman and Kampinga, 2009) and mutations in J-proteins are associated with highly tissue-specific diseases (Koutras and Braun, 2014; Sarparanta et al., 2020) such as early-childhood-onset recessive dilated cardiomyopathy and ataxia (Davey et al., 2006; Sparkes et al., 2007) and recessive distal hereditary motor neuropathy (Blumen et al., 2012).

Little is currently known regarding the mechanisms of how cells discretely modulate the expression of J-proteins in a tissue-specific manner or in response to stimuli or stress. Classically studied mechanisms of chaperone regulation are transcription factor activation (e.g., Heat Shock Factor 1) of a broad subset of chaperone gene targets during stress conditions such as heat shock (Zou et al., 1998; Anckar and Sistonen, 2011; Zheng et al., 2016) or ER-stress (Lee et al., 2003; Acosta-Alvear et al., 2007). Nonetheless, while some J-proteins exhibit stress-induced expression, most of the members of the family are constitutively expressed to cell or tissue specific levels (Zhao et al., 2008; Kakkar et al., 2012). In contrast to the stress response-activated transcription factors, even less is known about the post-transcriptional regulation of chaperone protein expression by other cellular factors such as microRNAs.

Considering the increasing understanding of J-proteins in protein folding-related diseases, such as the reported reduction of several J-proteins in Parkinson's Disease (Hasegawa et al., 2018), a more thorough understanding of the regulation of these proteins is warranted.

## MicroRNA-Mediated Silencing of mRNA Transcripts

Since the initial discovery of short non-coding RNAs regulating mRNA translation (Lee et al., 1993; Wightman et al., 1993; Reinhart et al., 2000), it has become apparent that microRNAs (miRNAs) function in the regulation of a large portion of the cellular transcriptome. It is estimated that each miRNA family targets on average more than 400 human mRNAs, and over half of human mRNAs have canonical conserved target sequences in their 3' untranslated regions (UTRs) (Friedman et al., 2009).

Endogenous miRNAs arise from long primary transcripts. A series of cellular processing events, depending on the transcript origin of the miRNA, produce the final mature miRNA in the Ago protein-containing silencing complexes (Bartel, 2018). The mature ~22 nucleotides (nt) miRNA, guide the Ago protein-containing complexes to their target mRNAs via base pairing. In canonical targeting of mRNAs, this involves contiguous base pairing of the 5' seed region of the miRNA (nts 2-7) (Bartel, 2018). Base pairing with additional 3' nucleotides in the miRNA can occur but has been reported to have minimal effects on silencing efficacy (Grimson et al., 2007; Wee et al., 2012; Salomon et al., 2015). While several mechanisms have been reported regarding the silencing of mRNAs by miRNAs, the repression mechanism dependent on the TNRC6 adaptor protein family is the dominant mechanism in humans, as recently reviewed and discussed (Jonas and Izaurralde, 2015; Bartel, 2018). In this mechanism, TNRC6 family proteins bind several miRNA-ago complexes and therefore enhance the silencing of several miRNAs to one mRNA.

## Current State of Reported miRNA Regulators of J-Proteins

While there have been numerous reports in the literature describing correlations of J-Protein expression with miRNA expression, there are relatively few examples where the target sequence in the 3' UTR of the J-Protein mRNAs has been experimentally validated (see Table 1). Reports only describing anti-correlations in J-Protein expression with miRNA expression were omitted as indirect regulator interaction networks cannot be ruled out without further investigation. Some of these excluded reports include examples with compelling data where miRNA-dependent regulation is through the 3' UTR of a target mRNA, such as the down regulation of DNAJC6 upon miR-146b-5p expression (Kirchmeyer et al., 2018). Cases where the target sequence for a miRNA was not verified were also excluded (Mitra et al., 2012; Yang et al., 2014; Mycko et al., 2015). Increasing complexity of regulation of long non-coding RNAs (lncRNA) (Goodall and Wickramasinghe, 2021) and their interactions with miRNA regulatory networks (López-Urrutia et al., 2019) could interfere in the miRNA-mRNA interactions in these cases. We will now briefly summarize verified microRNA targeting of J-proteins.

DNAJB1 is mostly known for the chimeric transcript it forms with PRKACA, which codes for the catalytic domain of protein kinase A in fibrolamellar hepatocellular carcinoma (Honeyman et al., 2014). It furthermore has been argued to be involved in p53-mediated apoptosis through degradation of PDCD5 (Cui et al., 2014). An investigation on a model for Spinocerebellar Ataxia Type 3 (SCA3) revealed a functional role for DNAJB1 in the clearance of mutant polyglutamine (polyQ) protein ataxin-3 aggregates. miR-370 and miR-543, which were both upregulated in SCA3 were shown to specifically target DNAJB1 mRNA. This study highlights possible disease implications miRNAs could have through their interactions with chaperones (Evert et al., 2018).

DNAJC3 is an ER-localized J-protein and co-chaperone to HSPA5. A loss-of-function mutation leads to diabetes mellitus and multisystemic neurodegeneration (Synofzik et al., 2015) and in mice, DNAJC3 knockout mice had a phenotype of partial loss of pancreatic beta-cells (Ladiges et al., 2005). In mouse models of beta cell stress and obesity, miRNA-200 family was found to have a role in promoting the apoptosis of pancreatic beta cells (Belgardt et al., 2015). Transcriptome analysis of miRNA-200 targets in mice revealed DNAJC3 which was then validated as a direct target.

DNAJB9 has recently been shown to inhibit p53-induced apoptosis (Lee H. J. et al., 2015). In models of chemotherapy resistance in acute myeloid leukemia, Wang et al. (2020) identified a regulatory network that involves the direct downregulation of DNAJB9 by miR-32. While miR-32 inhibited DNAJB9, it was in turn modulated by the lncRNA, small nucleolar RNA host gene 5 (SNHG5), creating an axis of control between DNAJB9-miR-32-SNHG5, possibly causing chemotherapy resistance. An analogous regulatory network has also been reported for DNAJB12, an ER-related J-protein (Ma et al., 2020), where the direct targeting of anti-apoptotic DNAJB12 by miR-152-3p is negatively modulated by the lncRNA HCG18 in gastric cancer models. Both studies highlight the complexity of gene regulation through the miRNA network, including the involvement of factors such as lncRNAs.

Additional evidence of miRNA targeting of J-Proteins can be taken where miRNA-Argonaute protein complexes have been identified to associate with the 3' UTRs. As an example, immunoprecipitation experiments revealed DNAJB11 as a component of miR-29-Ago complexes (Beitzinger et al., 2007). While this is strongly suggestive of a regulatory interaction, the authors indicate that not all the interactions identified lead to biological downregulation upon validation.

## MicroRNA Target Prediction of J-Proteins

While this list of validated regulatory interactions of miRNAs with J-proteins is quite limited, the correlative data in the literature suggests there are significantly more interactions awaiting validation.

To obtain a more global perspective on the potential miRNA network of interactions on the J-Proteins we performed an *in silico* analysis of miRNA target predictions. To this end, we utilized the most recent version of Targetscan (version 7.2) which identifies predicted canonical mRNA target sequences with 7–8 nt stretches of complementarity to the miRNA seed sequence (Agarwal et al., 2015).

Within the context of miRNA target prediction, both evolutionary conservation aspects of the miRNAs themselves and particular putative miRNA target sequences within a mRNA exist. With respect to our analysis, there are miRNAs and miRNA families that are broadly conserved among vertebrates and miRNAs conserved among mammals (Bartel, 2018). While there may be more recently evolved miRNAs, it has been proposed that many of these have too few targets under selective pressure to enable target predictions with any confidence (Friedman et al., 2009). A major caveat of any predicted miRNA target analyses is that not all mRNAs with 7–8 nt complimentary sites to the miRNA seed sequence exhibit regulation by that miRNA (Baek et al., 2008; Selbach et al., 2008).

**Figure 1:**
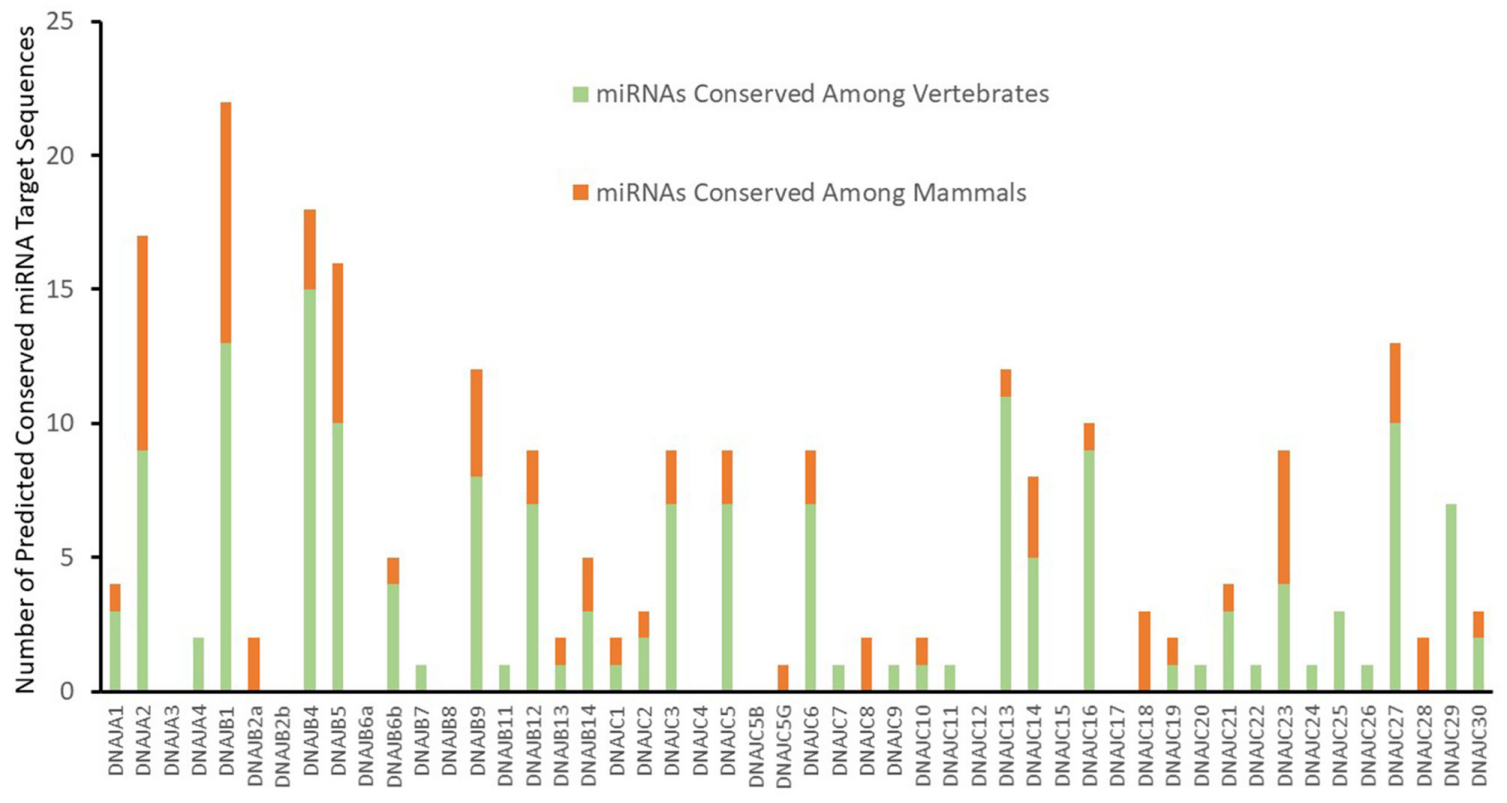
Predicted targeting by conserved miRNA Families. Predicted conserved J-Protein target sites of miRNAs from TargetScan (Agarwal et al., 2015) with a context score of <−0.1. The number of predicted conserved miRNA targets for each of the J-proteins are indicated. The proportion of predicted targets by miRNAs and miRNA families broadly conserved throughout vertebrates or only within mammals are also indicated.

An initial analysis for putative miRNA targets of the J-protein family with cumulative context scores of ≤−0.1 (Agarwal et al., 2015) as a first-pass threshold, results in 1,337 potential miRNA target sequences for 212 different miRNAs or miRNA families. This minimal criterion yields an unwieldy number of potential sites for experimental validation and most likely consists of a high proportion of false identifications. As mRNAs with evolutionary conserved potential miRNA target sequences in their 3' UTRs exhibit a higher probability of responding to the activity of a miRNA (Baek et al., 2008), strategies to identify more likely miRNA target sequences include choosing sites that exhibit conservation (Bartel, 2009; Friedman et al., 2009). As a result, we applied the criteria for evolutionarily conserved mRNA target sequences for both broadly conserved miRNAs among vertebrates and miRNAs conserved among mammals. This additional parameter reduces the likelihood of false positive identifications in the dataset, while including many possible cross-species interactions. Thus, although our additional analysis criteria decreases our false negative rate of prediction it will also miss some potentially biologically relevant chaperone-miRNA interactions that are not conserved among species. It should be noted that this new criteria also leads to the exclusion of target predictions for some highly probable targets. One example being the targeting of DNAJB5 by miR-21 (Lampis et al., 2018), where miR-21 expression was demonstrated to lead to 3' UTR dependent regulation of DNAJB5. This miR-21:DNAJB5 interaction was excluded from our presentation as a valid target as the predicted target sequence was not experimentally verified. With a selection criterion for conserved putative mRNA target sites for conserved miRNAs, Targetscan identifies 164 and 72 predicted targets respectively for either broadly conserved miRNAs or miRNAs conserved among mammals. This level of analysis reveals significant variations between the members of the J-protein family. As seen in Figure 1, nine mRNAs have no predicted canonical miRNA target sequences, such as DNAJA3, DNAJB8, and DNAJC4. On the opposite side of the spectrum, several J-Protein mRNAs contain a high number of predicted conserved miRNA targets. The mRNAs for DNAJA2, DNAJB1, DNAJB4, and DNAJB5 each have >15 predicted conserved miRNA target sequences within their 3' UTRs. Among these, only two of the predicted target sequences for DNAJB1 have been experimentally verified (Evert et al., 2018).

**Figure 2:**
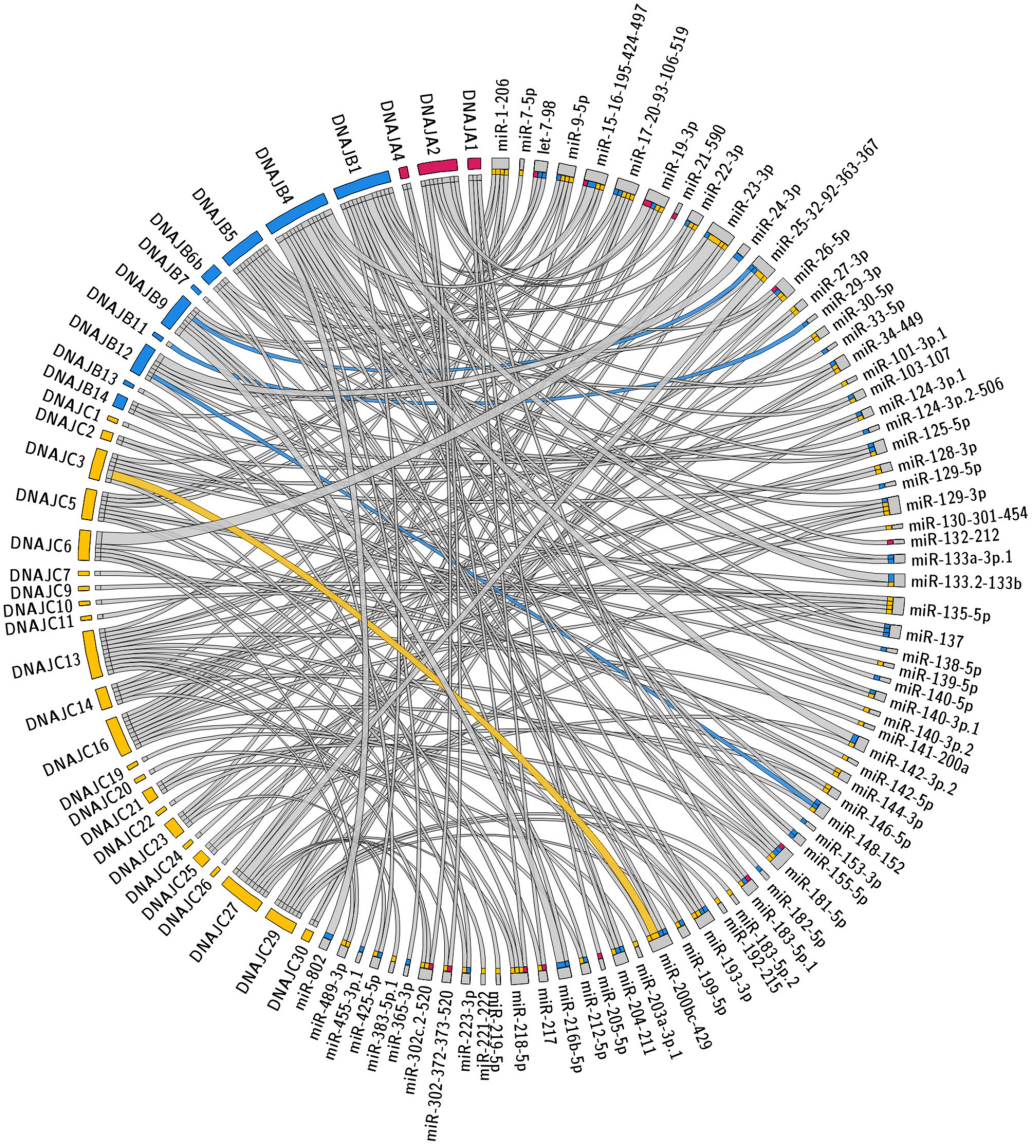
Predicted miRNA targets for miRNAs broadly conserved in vertebrates. Interaction Circos plot (Krzywinski et al., 2009) for TargetScan depicting targeting of J-Proteins by miRNA families that are broadly conserved throughout vertebrates. For each J-Protein, miRNAs with a context score of <−0.1 were extracted from TargerScan (Agarwal et al., 2015). Unconserved target sequences for miRNAs that were not conserved were excluded from the analysis. A colorized line indicates miRNA targeting that has been experimentally verified and the width of the bridging lines is proportional to the number of predicted target sequences.

**Figure 3:**
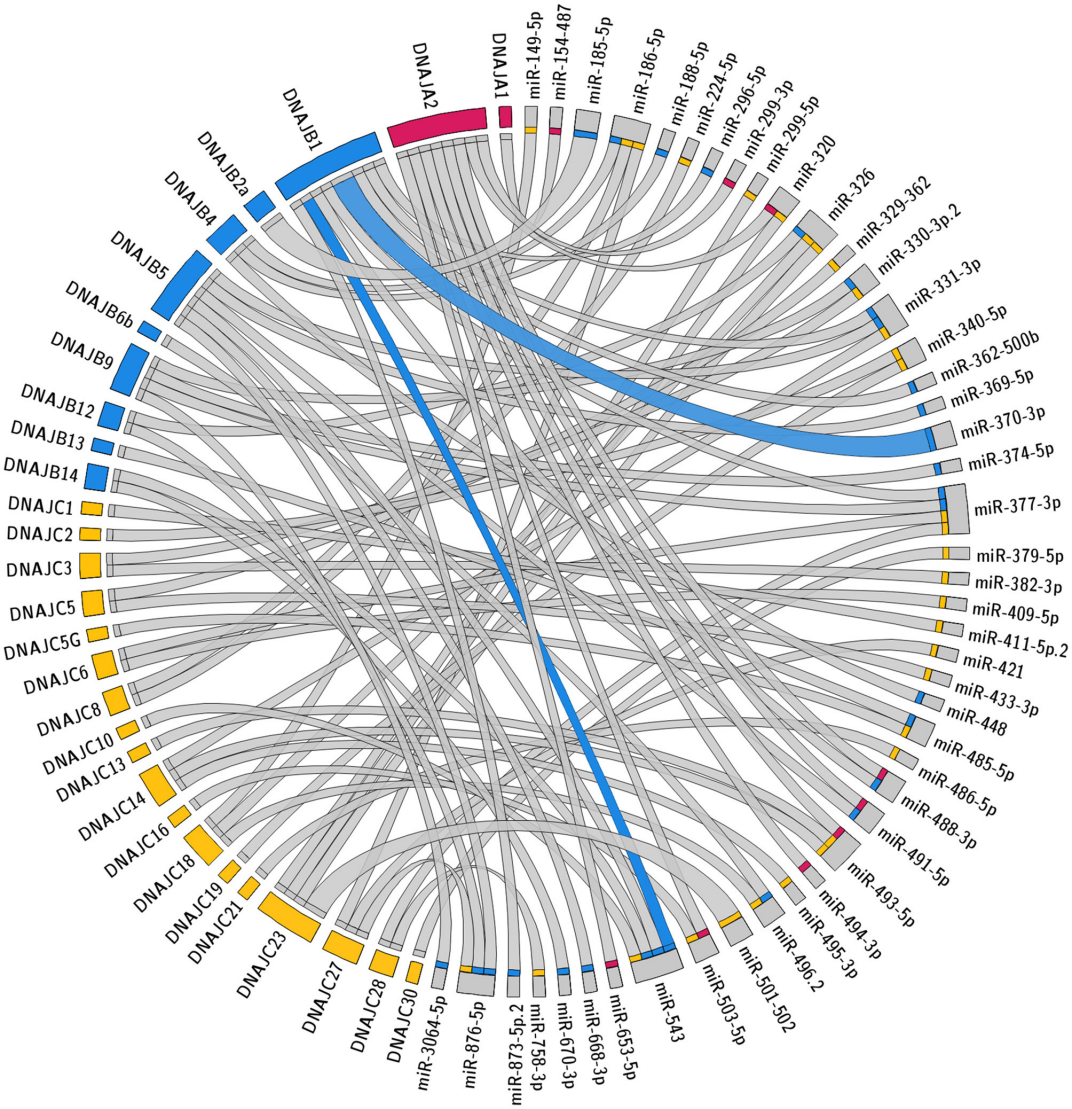
Predicted miRNA targets for miRNAs conserved within mammals. Interaction Circos plot (Krzywinski et al., 2009) for TargetScan depicting miRNA targeting of the J-Protein by miRNA families that are conserved throughout mammals. For each J-Protein, miRNAs with a context score of <−0.1 were extracted from TargerScan (Agarwal et al., 2015). Unconserved target sequences for miRNAs that were not conserved were excluded from the analysis. A colorized line indicates miRNA targeting that has been experimentally verified and the width of the bridging lines is proportional to the number of predicted target sequences.

The specific predicted conserved target interactions for all the J-Protein members by broadly conserved miRNAs or those conserved among mammals are shown in Figures 2, 3 respectively. With the reported known interactions listed in Table 1 highlighted in these figures, it is apparent how few of these predicted 234 interactions have been investigated. This therefore emphasizes an area of research in chaperone biology that is primed for further investigation.

### Predicting Strong miRNA Candidates of J-Protein Regulation

Despite the large number of predicted potential interactions, many predicted conserved miRNA target sequences are known to not significantly regulate expression levels of their targets in cells (Baek et al., 2008). Proteomic analysis of the same miRNAs in different cellular backgrounds reveals cell-specific differences (Ludwig et al., 2016; Piragasam et al., 2020) and changes in protein abundances that may counter interactions shown by miRNA target predictions (Piragasam et al., 2020). These proteomic analyses are holistic in that they reveal both direct and indirect impacts on protein abundance through miRNAs. Results that counter predictions can thus not be used to rule out potential interactions. Nonetheless, these types of analyses may provide some insight and guidance for predicting interactions that might lead to stronger regulation of a given target which can then be prioritized for investigation. In the next few paragraphs, we examine two potential miRNA mechanisms that may enhance their effects on target transcripts and highlight potential examples in our J-protein-miRNA dataset.

### Multiple miRNA Target Sites

The degree of regulation of mRNAs by miRNAs depends on the identities and abundance of the particular miRNAs in the cell, the number of target mRNA sites in a cell, as well as the specific binding efficacy for a given miRNA target site. For each given miRNA:mRNA interaction, the regulation is typically modest with repression being <50% (Baek et al., 2008; Selbach et al., 2008). However, enhanced repression is often observed when multiple miRNA target sites are within the same 3' UTR as these effects are typically additive. The predictions presented in Figures 2, 3 reveal several individual miRNAs that are predicted to have multiple mRNA target sequences within a given 3' UTR. These include the three predicted target sites for miR-23-3p within the 3' UTR of DNAJC6 and an additional 12 miRNA:mRNA interactions with two predicted miRNA target sites. Of the 12 predictions with two predicted target sites in an mRNA for the same miRNA, the targeting of DNAJB1 by miR-370 (Evert et al., 2018) and DNAJC3 by the miRNA-200 family (Belgardt et al., 2015) have been validated.

### Cooperative miRNA Targeting

Complex patterns of miRNA expression exist in different cell and tissue types (Landgraf et al., 2007; Chaulk et al., 2016). The intersection of both miRNA and mRNA expression patterns can yield increasingly complex combinatorial regulatory networks of regulation. This regulation becomes even more complex when considering that multiple miRNAs can simultaneously target the same 3' UTR of an mRNA and lead to differential outcomes for the same transcript depending on a cell's given miRNA signature pattern.

The potential for multiple different miRNAs targeting the same J-Protein is summarized in Figures 2, 3. Here it should be noted that the co-targeting of DNAJB1 by miR-543 and miR-370 has been documented (Evert et al., 2018). There is another level of complexity regarding co-targeting that is not revealed in the presentation of the figures, that is, their spatial proximity. In some cases, miRNAs are reported to act cooperatively on the same mRNA, specifically those with target sequences within 8–40 nt of each other (Grimson et al., 2007; Sætrom et al., 2007). This is a result of the TNRC6 proteins being able to associate with multiple Ago protein complexes simultaneously (Briskin et al., 2020).

**Figure 4:**
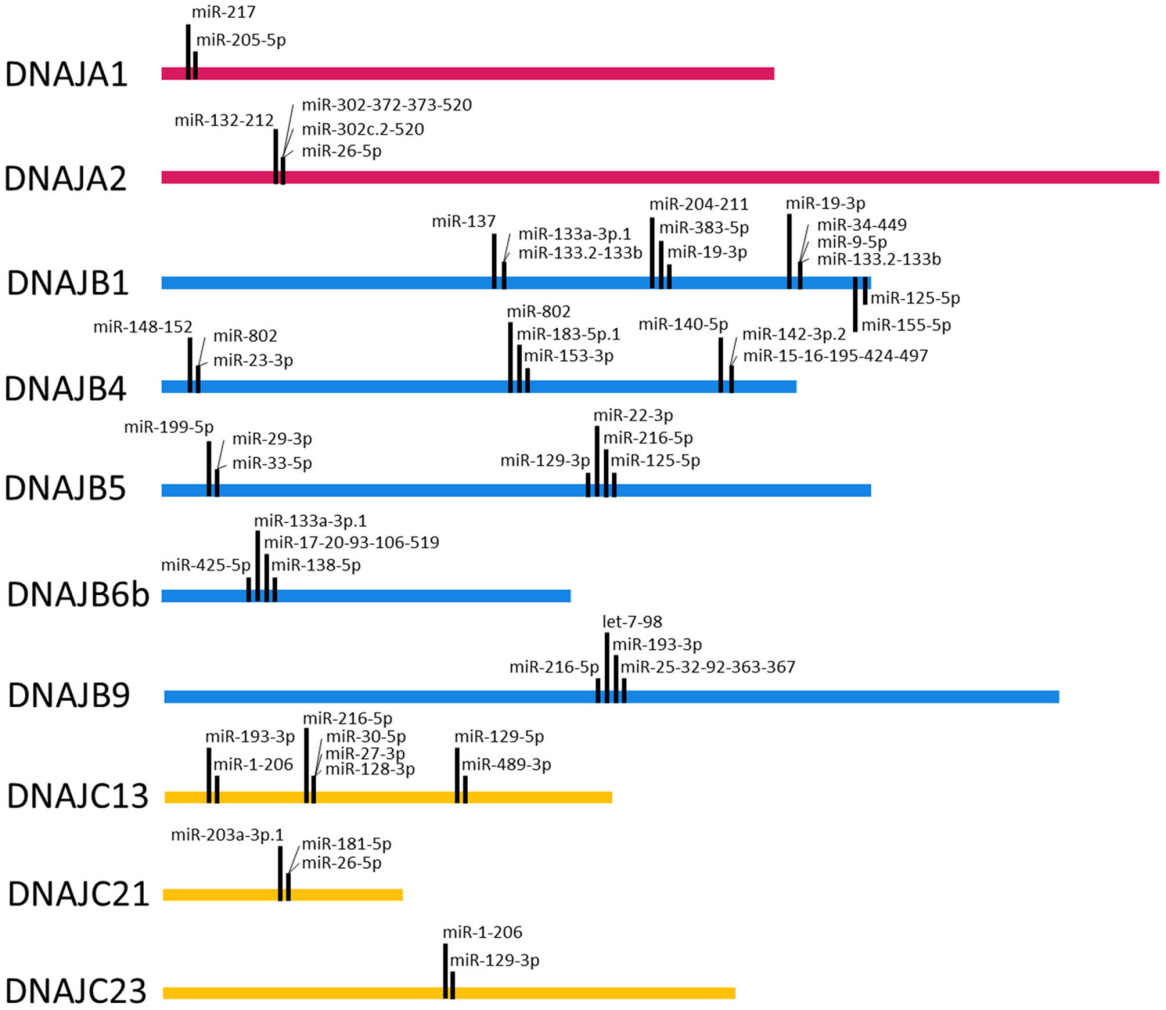
Target Site Proximity. For each J-Protein, target sites were identified on TargetScan (Agarwal et al., 2015) with a context score of <−0.1 and then manually scanned for the occurrence of miRNA seed sequences <40 nucleotides apart. The locations of identified close proximity target sites are visualized on the ribbon corresponding to the nucleotide length of the 3' UTR for each given J-Protein. Only miRNA families that are broadly conserved through vertebrates were included in the analysis.

To query the potential for cooperative interactions between the predicted miRNA target sequences in the 3' UTRs of the J-protein mRNAs, target sequences within 8–40 nts of each other were identified. Figure 4 depicts the miRNA target sequences that meet this criterion for the miRNA families broadly conserved among vertebrates. Intriguingly, the analysis reveals a potential for another level of complexity with regard to miRNA regulation. While the analysis identified multiple examples of potential miRNA target sequences within 8–40 nts, such as the predicted miR-217 and miR-205-5p target sequences in the 3' UTR of the DNAJA1 mRNA, there are also potential combinations of mutual exclusivity. For example, as shown in Figure 4, the DNAJB4 mRNA has predicted miRNA target sequences for the miR-148-152 family within 40 nts of the predicted target sequences for miR-802 and miR-23-3p. As the predicted target sequences for miR-802 and miR-23-3p partially overlap, then if both sequences exhibit bonafide mRNA targeting in cells, they would have to be mutually exclusive in their targeting by these miRNAs. This leads to the prediction that miR-148-152 could act cooperatively with either miR-802 and miR-23-3p but that these two miRNAs could not bind to the same DNAJB4 mRNA to regulate its expression. While this form of potential regulation leads to numerous instances of a Boolean logic type of regulation behavior, it is currently unclear whether this behavior would be recapitulated in cells and would be highly dependent on miRNA complex concentrations.

## Conclusion

Perhaps as a result of the enormous inherent complexities of both the chaperone networks and miRNA regulatory networks, there has been relatively little reported work validating their intersection of regulation. Our analysis offers starting points for the exploration of miRNA and J-protein interactions. With the reported linkages and interest in both miRNAs and J-proteins in human diseases such as neurodegeneration and cancer, there are ample possibilities that we have outlined for future investigations into the interplay of these systems.

## Author Contributions

LB: drafted the manuscript and collected literature and data. RF: data analysis and edited the manuscript. SAM: designed the content of the article and edited the manuscript. All authors contributed to the article and approved the submitted version.

## Conflict of Interest

The authors declare that the research was conducted in the absence of any commercial or financial relationships that could be construed as a potential conflict of interest.
